# Validation of existential fulfillment scale in Chinese university students

**DOI:** 10.3389/fpsyg.2024.1497422

**Published:** 2025-01-16

**Authors:** Jian Chen, Xiaoyan Chen, Chen Miao

**Affiliations:** School of Psychology, Fujian Normal University, Fuzhou, Fujian, China

**Keywords:** existential fulfillment, reliability, validity, Chinese university students, confirmatory analysis (CFA)

## Abstract

**Introduction:**

This study aimed to assess the psychometric properties of the Existential Fulfillment Scale (EFS) in a Chinese university student sample, emphasizing the cultural fit of the scale.

**Methods:**

A cohort of 1,600 undergraduate students from six universities in Fujian Province completed questionnaires including the EFS, Meaning of Life Questionnaire (MLQ), Index of Well-Being (IWB), and Self-Depression Scale (SDS). We conducted item analysis, exploratory factor analysis (EFA), confirmatory factor analysis (CFA), and assessments of criterion-related validity, internal consistency, and test-retest reliability.

**Results:**

The Chinese EFS consists of two dimensions—self-acceptance and self-breakthrough—across 14 items, reflecting cultural distinctions from the original model by combining the dimensions of self-actualization and self-transcendence. This revised structure aligns with Chinese cultural perspectives on individual growth, where self-actualization often integrates aspects of self-transcendence. The scale showed positive associations with the MLQ and IWB and a negative association with the SDS, supporting the scale’s criterion-related validity. Internal consistency ranged from 0.87 to 0.97, and test-retest reliability ranged from 0.75 to 0.83.

**Discussion:**

These findings indicate that the Chinese EFS is a reliable tool for assessing existential fulfillment among Chinese university students.

## 1 Introduction

### 1.1 Conception of existential fulfillment

Existential fulfillment is a central construct in existential psychology and stands as a foundational element of Viktor Frankl’s theory of the “Existential Vacuum” and logotherapy (Loonstra et al., 2007). Frankl (1963) described the existential vacuum as a condition marked by an absence of meaning and purpose, which leads individuals to experience profound unease, emotional detachment, and a pervasive sense of life’s meaninglessness (Frankl, 1963; Yalom, 1980). This existential void is often accompanied by feelings of insecurity and desolation, as individuals struggle to find direction.

In contrast to the existential vacuum, existential fulfillment embodies a life enriched with significance and purpose (Längle et al., 2003; Loonstra et al., 2007). Frankl (1962) argued that the search for meaning is the most profound motivational force for humans, achieved through the pursuit and embodiment of life’s inherent values. This drive, termed the “will to meaning,” reflects humanity’s inherent quest to realize their potential and discover value in their existence (Frankl, 1962). Expanding on this, Rollo May introduced the concept of a “sense of existence” or “sense of being,” emphasizing the experiential dimension of existential fulfillment. May’s approach highlighted key characteristics of existential experience: self-centering, self-affirmation, engagement, awareness, self-consciousness, and anxiety (May et al., 1958; Yang, 1999).

Clinical and empirical research in psychology consistently highlights the central importance of existential fulfillment, recognizing it not only as a core aspect of personality but also as a crucial determinant of mental health. A wealth of studies have demonstrated strong correlations between existential fulfillment and various indicators of psychological well-being, including emotional stress levels, neuroticism, burnout, work engagement, internet addiction, vitality and self-awareness of health (Tomic and Tomic, 2008; Mausch and Rys, 2020; Längle, 2011; Längle et al., 2003; Tomic and Tomic, 2011; Loonstra et al., 2010; Levchenko, 2024; Mikhaylova and Dmitrieva, 2021; Riethof and Bob, 2019). Individuals with a strong sense of existential fulfillment exhibit an enhanced capacity for value-based decision-making, personal goal setting, and extracting meaning from life (Yang, 1999). Conversely, those experiencing an existential vacuum face a heightened risk of mental health challenges, such as depression, anxiety, and despair, which can lead to a loss of creativity, freedom, and willpower, and, in severe cases, contribute to disorders such as schizophrenia (Längle et al., 2003; Frankl, 1962; Ratcliffe, 2009). Echoing these sentiments, Rollo May posits that the erosion of existential fulfillment is central to mental illness, advocating for its restoration as a primary aim in psychotherapy (May et al., 1958; Yang, 1999).

### 1.2 Measurement of existential fulfillment

Drawing upon the foundational principles of Viktor Frankl’s logotherapy and the concept of existential vacuum, Längle et al. (2003) initially defined existential fulfillment as a multidimensional construct, encompassing fundamental elements of human reality: self-distance (perception), self-transcendence (value recognition), freedom (decision-making), and responsibility (acting). The Existence Scale (ES), developed by Längle et al. (2003), was designed to assess these existential competencies, evaluating individuals’ capacity to engage meaningfully with themselves and the world.

The ES comprises 46 items across four subscales—self-distance, self-transcendence, freedom, and responsibility—and utilizes a six-point Likert scale ranging from “fully disagree” to “fully agree.” Although its theoretical foundation was robust, confirmatory factor analysis (CFA) did not confirm the ES’s construct validity, as Brouwers and Tomic (2012) observed. This limitation sparked renewed interest in refining the conceptualization and measurement of existential fulfillment, prompting further development of assessment tools (Loonstra et al., 2007).

Prominent among these scholarly pursuits, Loonstra et al. (2007) made a significant contribution to existential psychology by refining and clarifying the concept of existential fulfillment, which they identified as an essential aspect of a meaningful and purpose-driven lifestyle. Their conceptualization, deeply rooted in humanistic-existential psychology, integrates the philosophical principles of Viktor Frankl’s logotherapy, Irvin Yalom’s existential psychotherapy, the humanistic ideals of Abraham Maslow, Carl Rogers, and the existential humanism of Erich Fromm. This unified theoretical framework defines existential fulfillment as a life goal that fully honors the nature of human existence, acknowledging three fundamental limitations: the finality imposed by mortality, the boundaries of individual potential, and the constraints shaped by relationships with others and the larger world. To navigate these intrinsic limitations and achieve fulfillment, individuals are tasked with meeting three core existential needs: self-acceptance, self-actualization, and self-transcendence.

The fulfillment of these three tasks is thought to culminate in existential fulfillment. According to the authors, self-acceptance involves a wholehearted embrace of one’s reality, including the acceptance of mortality, personal limits, and the recognition of one’s small role in a vast reality. Self-actualization is the process through which an individual realizes their intrinsic value and authentic potential. Self-transcendence, central to mental health, represents the cognitive ability to engage meaningfully with both one’s inner self and the external world (Loonstra et al., 2007).

To effectively measure existential fulfillment, a scale must meet two criteria. First, it should assess the degree to which people live with purpose and meaning, reflecting the nature of existential fulfillment as a life goal. Second, the scale should differentiate between various qualitative types of values, goals, and life meanings, distinguishing between extrinsic and intrinsic, as well as self-centered and self-transcending, aspirations (Loonstra et al., 2007). Existing scales, such as the Purpose in Life Test (PLT; Crumbaugh, 1968), Life Regard Inventory (LRI; Battista and Almond, 1973), and Meaning in Life Scale (MLS; Steger et al., 2006), satisfy the first criterion by assessing life’s purpose and meaning. However, they fall short of distinguishing between self-alienated, self-actualizing, and self-transcending values, thereby failing to meet the second criterion (Loonstra et al., 2007). Hence, a new scale was deemed necessary.

Recognizing these considerations and drawing on an advanced, holistic understanding of existential fulfillment, Loonstra et al. (2007) developed the Existential Fulfillment Scale (EFS). The EFS encapsulates three core constructs of existentialism—self-acceptance, self-actualization, and self-transcendence—each representing distinct dimensions of existential fulfillment. Comprising 15 items, with five items for each dimension, the EFS is well-structured to assess these aspects. The CFA showed that, compared to four alternative models, the three-factor oblique model yielded the best fit, with a Root Mean Square Residual (RMR) of 0.08, Goodness of Fit Index (GFI) of 0.91, Adjusted Goodness of Fit Index (AGFI) of 0.88, Normed Comparative Fit Index (CFI) of 0.89, and Parsimony Normed Comparative Fit Index (PCFI) of 0.73. Additionally, significant correlations emerged between self-acceptance and self-actualization (*r* = 0.41, *p* < 0.001) and between self-actualization and self-transcendence (*r* = 0.40, *p* < 0.001), though no significant correlation was observed between self-acceptance and self-transcendence (*r* = 0.06, *p* > 0.05). These results have been corroborated by subsequent studies (Loonstra et al., 2010; Tomic and Tomic, 2011; Bekenkamp et al., 2014; Kay, 2014, 2019). Overall, the study supports the presence of three distinct but related dimensions within existential fulfillment, with internal consistency coefficients for self-acceptance, self-actualization, and self-transcendence at 0.74, 0.71, and 0.88, respectively.

However, the study did not examine several critical indicators of validity, including convergent, discriminant, and criterion-related validity, nor did it assess stability through test–retest reliability, which the authors acknowledge as a limitation. Furthermore, they recommend that future studies theoretically explore and empirically investigate the interrelationships among the three constructs of existential fulfillment in greater depth (Loonstra et al., 2007).

Despite these limitations, the EFS has become widely accepted in empirical research on existential fulfillment, effectively replacing the Existence Scale (ES) in many studies (Loonstra et al., 2010; Tomic and Tomic, 2011; Bekenkamp et al., 2014; Kay, 2014, 2019; Tomic, 2016). The scale has consistently demonstrated strong reliability and validity across multiple studies and has been validated and applied in various cross-cultural contexts, underscoring its broad acceptance and esteem within the research community.

### 1.3 Existential fulfillment, well-being and depression

Existential fulfillment plays a crucial role in psychological health and life satisfaction, forming an integral part of an individual’s overall well-being (Tomic and Tomic, 2011). Frankl’s (2004) logotherapy underscores the centrality of the quest for meaning in human life, suggesting that achieving existential goals can foster fulfillment and enhance well-being. Tomic and Tomic’s (2011) findings support this, identifying a positive correlation between self-transcendence—a dimension of existential fulfillment—and well-being, particularly in professions with high interpersonal demands, such as nursing. Similarly, Bekenkamp et al. (2014) observed that elevated levels of self-actualization and self-acceptance contribute to better perceived physical health, a significant correlate of overall well-being. Collectively, these studies suggest that existential fulfillment serves as a protective factor against psychological distress and is a vital contributor to well-being.

Conversely, existential fulfillment is inversely related to depression, with a lack of meaning and purpose often preceding depressive symptoms and burnout (Shek et al., 2022; Alfuqaha et al., 2021). Mascaro and Rosen (2008) found that a sense of existential meaning could predict lower depressive symptoms over time, while Längle et al. (2003) and Loonstra et al. (2010) demonstrated that higher levels of existential fulfillment were associated with reduced depression, especially in high-stress fields such as teaching. Thakur and Basu (2010) also identified a significant correlation between depression and existential meaning, suggesting that promoting fulfillment may be an effective strategy for both preventing and alleviating depressive symptoms. These findings underscore the value of existential dimensions in both the understanding and treatment of depression.

### 1.4 The present study

China’s rapidly evolving socio-economic landscape has led to transformative shifts, moving beyond material scarcity and prompting heightened spiritual and existential inquiries among its people. This trend has given rise to various psychological challenges, including feelings of emptiness, a lack of purpose, and diminished spiritual direction (Wu, 2018; Zhang, 2018), thus intensifying the relevance of existential concerns in contemporary mental health discourse. Given this context, investigating the existential psychology of Chinese individuals has taken on both an urgent and a practical importance. However, empirical research into these existential challenges in China remains sparse, with few studies specifically addressing existential fulfillment. To address this gap, we propose that adopting and adapting a relevant existential fulfillment scale could be a critical first step in advancing research in this field. Considering the dual priorities of reliability and validity, the EFS emerges as a well-suited instrument for revision to meet the specific demands of this research. Thus, this study aims to assess the psychometric properties of the EFS and evaluate its applicability within a sample of Chinese college students, who serve as a representative demographic for this research.

Our objectives are twofold: first, to validate the factorial structure of the EFS through item analysis and exploratory factor analysis (EFA); second, to establish the factorial validity of the EFS in alignment with previous research, confirm its criterion-related validity, and assess its reliability through internal consistency and test–retest stability.

## 2 Materials and methods

### 2.1 Participants

Samples 1 and 2 were utilized for comprehensibility testing. Sample 1 included 25 undergraduate students from a university in Fujian Province, with 6 freshmen, 6 sophomores, 5 juniors, and 8 seniors. This sample comprised 12 male and 13 female students, with a mean age of 20.18 years (*SD* = 1.98). Sample 2 matched this composition and was drawn from the same institution, consisting of 7 freshmen, 8 sophomores, 5 juniors, and 5 seniors, including 11 male and 14 female students, with a mean age of 20.02 years (*SD* = 1.81).

Sample 3 was designated for Item Analysis and EFA. This group comprised undergraduates from six universities in Fujian Province, recruited via a stratified cluster sampling method based on academic disciplines, gender, and academic level. Of the 380 questionnaires distributed, 324 valid responses were returned, yielding a response rate of 85.3%. This sample included 141 males and 183 females, with a mean age of 20.31 years (SD = 2.11). Academic standings included 95 freshmen, 83 sophomores, 75 juniors, and 71 seniors, representing diverse fields: 111 students from the Arts, 101 from Science and Technology, and 112 from other disciplines.

Sample 4 was used for CFA and internal consistency test. This sample, drawn again from undergraduates across the same six universities, was collected via stratified cluster sampling to reflect academic major, gender, and year. Of the 800 questionnaires distributed, 727 valid responses were collected, achieving a response rate of 90.9%. The sample included 321 males and 406 females, with a mean age of 20.67 years (*SD* = 1.98), and covered 171 freshmen, 173 sophomores, 195 juniors, and 188 seniors across various disciplines: 241 students from the Arts, 276 from Science and Technology, and 210 from other fields.

Sample 5 and Sample 6 were used to assess criterion-related validity. Sample 5 included 317 students (131 male, 186 female, mean age = 21.03 years, *SD* = 1.62) from a university in Fujian Province, who completed the EFS, Meaning of Life Questionnaire (MLQ), and Index of Well-Being (IWB). Sample 6 comprised 33 students (15 male, 18 female, mean age = 20.84 years, *SD* = 1.25), who completed the EFS and the Self-Depression Scale (SDS).

Sample 7 was used to evaluate test–retest reliability, including 149 undergraduates (61 male, 88 female, mean age = 20.67 years, *SD* = 1.24) from a university in Fujian Province. Participants completed the questionnaire twice over a two-week period.

Each of these samples was independently recruited, with no overlap among participants. Samples 1–7 were initially utilized for the original analyses as described previously. However, to maximize the utility of these samples and provide additional evidence of validity and reliability, cross-validation analyses were subsequently performed as follows:

- CFA was first conducted on Sample 4 as the primary analysis, followed by CFA on Sample 5 to provide additional structural validity evidence.
- Internal consistency reliability analysis was initially conducted on Sample 4 and was later extended to Samples 5 and 7 to enhance the robustness of reliability evidence.

### 2.2 Measures

#### 2.2.1 Existential fulfillment scale (EFS)

The EFS, developed by Loonstra et al. (2007), includes 15 items designed to measure key dimensions of existential fulfillment across three factors: self-acceptance, self-actualization, and self-transcendence. Responses are captured on a 5-point Likert scale ranging from 1 (“not at all”) to 5 (“fully”), with higher scores indicating greater existential fulfillment.

#### 2.2.2 Meaning of life questionnaire (MLQ)

The MLQ, initially developed by Steger et al. (2006) and subsequently translated and revised by Wang and Dai (2008), was employed in this study. Comprising 10 items, the MLQ captures two core dimensions: the pursuit of meaning (seeking) and the presence of meaning in life (experiencing). In this study, the internal consistency reliability coefficients for the total scale, as well as the two subscales for seeking and experiencing meaning, were 0.84, 0.68, and 0.69, respectively, indicating a satisfactory level of reliability.

Since the EFS draws on Viktor Frankl’s logotherapy framework, as elaborated by Loonstra et al. (2007), it was hypothesized that existential fulfillment would conceptually overlap with the sense of meaning in life. Consequently, the MLQ scores were included as a criterion-related validity indicator, with the expectation of a positive correlation between the MLQ and EFS scores.

#### 2.2.3 Index of well-being (IWB)

The IWB, originally created by Campbell et al. (1976) and adapted by Wang et al. (1999), was also utilized in this study. This scale consists of two components: a general affective index with 8 items and a life satisfaction index comprising a single item. The overall score is computed by combining the average score of the Overall Emotionality Index with the Life Satisfaction Index score, which is weighted at 1.1 to reflect its relative importance. Previous studies report a test–retest reliability of 0.849 for the IWB (Wang et al., 1999). In this study, the internal consistency of the total scale and the general affective index were 0.82 and 0.87, respectively, indicating strong reliability.

Clinical and empirical research in psychotherapy consistently links existential fulfillment with subjective well-being (Tomic and Tomic, 2008; Mausch and Rys, 2020). Thus, IWB scores were incorporated to assess the criterion-related validity of the EFS, with a hypothesized positive correlation between these two scales.

#### 2.2.4 Self-depression scale (SDS)

The SDS, originally developed by Zung and cited in Wang et al. (1999), was used in this study as a measure of depressive symptoms. This 20-item scale is organized into four dimensions: psychotic-affective symptoms (2 items), somatic disorders (8 items), psychomotor disorders (2 items), and psychological disorders of depression (8 items). The SDS evaluates the severity of depressive symptoms experienced by participants over the preceding week. Internal consistency reliability for the total scale and its four subscales in this study were 0.79, 0.77, 0.72, 0.74, and 0.67, respectively.

In line with Frankl’s (1962) logotherapy, which positions the “existential vacuum” as an antithesis to existential fulfillment and associates it with depressive symptoms, the SDS was included as a criterion-related validity measure for the EFS. It was hypothesized that EFS scores would negatively correlate with SDS scores, reflecting the inverse relationship between existential fulfillment and depressive symptoms.

### 2.3 Procedure

#### 2.3.1 Data collection

The study protocol was approved by the Ethical Review Board of the School of Psychology at Fujian Normal University (Protocol No. Psy240051). Samples 1 and 2 were collected for comprehensibility testing by recruiting students from the researcher’s institution to complete an online questionnaire. For Samples 3 through 8, participants were independently recruited from six universities across Fujian Province through collaborations with faculty members who facilitated student recruitment in their respective classes. Each sample was recruited separately, ensuring there was no overlap among participants.

To strengthen the validity and generalizability of the findings, independent samples were designated for each stage of analysis, including item analysis, EFA, CFA, criterion-related validity and reliability testing. This approach reduced potential biases from overlapping samples and enhanced the reliability of the scale’s validation across different datasets. By using distinct samples for structural validation and criterion-related validity assessments and reliability testing, each analysis phase could contribute independently to a comprehensive evaluation of the scale’s psychometric properties.

Drawing on prior experience, the research team anticipated that including too many items in a single questionnaire could lead to fatigue or irritability among Chinese college students, potentially affecting response quality. Therefore, data collection was conducted in phases, aligning with each analytical objective to optimize data validity. For instance, during the CFA phase, participants were asked only to complete the EFS, minimizing respondent fatigue and enhancing data accuracy for structural examination.

Initially, the MLQ and IWB were selected as criterion measures to assess the criterion-related validity of the EFS. However, after analyzing data from these scales, the strong relationship between existential fulfillment and mental health became apparent. To further reinforce the criterion-related validity of the EFS, the SDS was included as an additional criterion, prompting the recruitment of Sample 6 specifically for this purpose.

#### 2.3.2 Translation of instrument

Dr. Welko Tomic, one of the original authors of the EFS, authorized the adaptation of the scale for use in Chinese. The translation process followed rigorous standards to ensure fidelity to the original English version, as outlined by the International Test Commission (ITC) guidelines^1^. In this study, the EFS was translated into Simplified Chinese and then back-translated into English through a multi-stage process designed to ensure conceptual, semantic, and cultural equivalence. Initially, a bilingual psychology researcher with a year of study experience in the United States translated the English version into Simplified Chinese, carefully preserving the meaning of the original items. Another bilingual psychology researcher, holding a Ph.D. from a U.S. university, then back-translated the Chinese version into English. Both translators were native Chinese speakers proficient in English, ensuring linguistic accuracy and cultural sensitivity. To assess conceptual equivalence, a panel of psychology experts familiar with existential fulfillment constructs in both Chinese and Western contexts reviewed the translated items. This review ensured that the items retained the underlying constructs of the original scale. Semantic equivalence was further tested with a pretest involving 25 bilingual participants, who rated the clarity and similarity of meaning between the English and Chinese items. Discrepancies were resolved through consultation with the translators. Cultural relevance was validated by consulting Chinese psychologists experienced in cross-cultural research, who reviewed each item for appropriateness in the Chinese university student context. The English back-translation was then sent to Dr. Tomic, who confirmed its accuracy, endorsing the adapted scale for use in the Chinese context.

An initial comprehensibility assessment was subsequently conducted. A sample of 25 university students from various academic disciplines (Sample 1) was asked to rate the clarity of each translated item on a 5-point Likert scale, where 1 indicated “difficult to comprehend” and 5 represented “fully comprehensible.” Participants were encouraged to explain any item rated below 4 to clarify areas needing improvement. Results revealed that items 1, 10, and 13 scored below 4, with comments indicating that these items were “not easy to understand” or “too broad.” Based on this feedback, the research team implemented targeted revisions to improve clarity.

A second comprehensibility test was conducted after these revisions. A new cohort of 25 college students, representing a range of academic majors, genders, and grade levels (Sample 2), assessed the scale’s clarity using the same 5-point Likert scale. This time, no complaints were raised regarding the phrasing, and all 15 items scored 4 or above, indicating high understandability.

Finally, the finalized Chinese version of the EFS, with response options identical to the original scale, was established for use in this study.

### 2.4 Data analysis

Initially, item analysis for the EFS was conducted using corrected item-total correlations, following the methodological approach outlined by Wu (2010).

Subsequently, EFA of the EFS was performed using SPSS software, version 19.0. The factor structure of the EFS items was determined through Principal Axis Factoring analysis, followed by Promax rotation to enhance factor interpretability.

To assess construct validity, CFA of the Chinese EFS was conducted using Mplus software, version 8.3. This analysis aimed to confirm that the indicators measured corresponded to three underlying latent factors. Established criteria for an adequate model fit were applied, targeting a CFI and Tucker–Lewis Index (TLI) of 0.90 or above, and a Root Mean Square Error of Approximation (RMSEA) and Standardized Root Mean Square Residual (SRMR) of 0.08 or below, in line with methodological guidelines provided by Byrne (1994) and Hu and Bentler (1995).

The criterion-related validity of the Chinese EFS was subsequently examined using the Chinese versions of the MLQ, IWB, and SDS.

Finally, the internal consistency of the Chinese EFS was evaluated by calculating the composite reliability, with higher values indicating greater internal consistency. Additionally, the test–retest reliability of the EFS was assessed with a two-week interval to confirm temporal stability.

## 3 Results

### 3.1 Item analysis

An item analysis was conducted on data from 324 participants in Sample 3. The analysis indicated that Item 8 had a total correlation coefficient of 0.35, which fell below the threshold of 0.40. Consequently, in accordance with the screening criteria outlined by Wu (2010), this item was excluded. The remaining 14 items showed correlation coefficients above 0.40, ranging from 0.41 to 0.76, and were statistically significant.

Next, participants were subsequently divided into high and low groups based on the upper and lower 27% of their total scores. A Multivariate Analysis of Variance (MANOVA) with a Bonferroni correction was applied to compare two groups. The results indicated a significant multivariate effect for group, with Pillai’s Trace, Wilks’ Lambda, Hotelling’s Trace, and Roy’s Largest Root all yielding a significant *F*-value of 57.99, df = 14, 186, *p* < 0.001, and a large effect size (Partial Eta Squared = 0.81). This suggests that the group variable significantly influenced the scores on the items.

Subsequent pairwise comparisons, adjusted for multiple comparisons using the Bonferroni method, revealed significant mean differences for all 14 items between the two groups. Each item demonstrated a statistically significant difference at the 0.05 level, with all *p*-values being less than 0.000, indicating a strong and consistent pattern of group differences across all items. The mean differences ranged from 0.78 to 1.44, with standard errors varying between 0.10 and 0.14, and all 95% confidence intervals excluding zero, confirming the significance of the observed differences.

In summary, the MANOVA and Bonferroni-adjusted pairwise comparisons provided robust evidence that the two groups significantly differed on all 14 items, affirming each item’s discriminative capability.

### 3.2 Exploratory factor analysis (EFA)

Exploratory factor analysis was conducted using data from Sample 3 (Table 1). The Kaiser–Meyer–Olkin (KMO) measure was 0.840, and Bartlett’s test of sphericity yielded a chi-square value of 1258.319 (*p* < 0.001), confirming the data’s suitability for factor analysis. A Principal Axis Factoring analysis with Promax rotation identified three factors with eigenvalues greater than 1 (1.13, 1.89, and 4.43), explaining 53.22% of the variance, which is close to the acceptable threshold of 50% (Wu, 2010). Factor loadings ranged from 0.48 to 0.79, with all items meeting the communality criterion (≥0.4). Inter-factor correlations were 0.53 between Factors 1 and 2, 0.32 between Factors 1 and 3, and 0.64 between Factors 2 and 3.

**Table 1:** Standardized factor loadings of the Chinese EFS.

Item	Item content	Factor 1	Factor 2	Factor 3
1	I often feel uncertain about the impression I make on other people	**0.49**	0.22	0.35
3	I do a lot of things that I would actually rather not do	**0.67**	0.31	0.16
11	I find it very hard to accept myself	**0.48**	0.43	0.35
12	I often do things because I have to, not because I really want to do them	**0.73**	0.30	0.16
4	I feel incorporated in a larger meaningful entity.	0.27	**0.60**	0.32
5	Deep inside I feel free.	0.34	**0.60**	0.43
6	I think I am part of a meaningful entity.	0.40	**0.79**	0.45
7	Even in busy times I experience feelings of inner calmness.	0.33	**0.54**	0.43
2	I’ll remain motivated to carry on even in times of bad luck.	0.25	**0.50**	0.50
9	It is my opinion that my life is meaningful.	0.43	0.55	**0.74**
10	I have experienced that there is more in life than I can perceive with my senses.	−0.01	0.17	**0.49**
13	I think my life has such a deep meaning that it surpasses my personal interests.	0.02	0.34	**0.63**
14	I completely approve of the things that I do	0.33	0.49	**0.62**
15	My ideals inspire me.	0.25	0.54	**0.73**

Factor 1 = self-acceptance; Factor 2 = self-actualization; Factor 3 = self-transcendence. Bolded values in the table indicate the factor loadings for items that are ultimately assigned to each of the three factors after the EFA.

Two notable discrepancies emerged when comparing the adapted scale to the original. First, the self-acceptance dimension was reduced to four items after the exclusion of Item 8. Second, some items were reassigned: Item 4 (“I feel incorporated in a larger meaningful entity”) and Item 6 (“I think I am part of a meaningful entity”), originally under self-transcendence, were moved to self-actualization, while Item 14 (“I completely approve of the things that I do”) and Item 15 (“My ideals inspire me”), initially categorized as self-actualization, were shifted to self-transcendence. Despite these adjustments, the factors retained their original names, aligning with the theoretical basis of the scale. These adjustments are discussed further in the discussion section.

Additionally, several items in Factors 2 and 3, such as Items 2, 5, 6, 7, 9, 14, and 15, had loadings above 0.4 on both factors. Item 5 showed equal loadings of 0.50 on both, raising the question of whether these could be considered a single factor, which will be explored in the CFA.

### 3.3 Confirmatory factor analysis (CFA)

The CFA was conducted using Mplus version 8.3 on a dataset of 727 valid responses from Sample 4, with parameters estimated via robust maximum likelihood (MLR) estimation. Following the original structural framework of the EFS, a three-factor model (M3) was created to represent the dimensions of self-acceptance, self-actualization, and self-transcendence. To enable comparative analysis, two alternative models, M1 and M2, were also tested.

M1 was a single-factor model where all items load onto a single construct. This model was tested to explore whether existential fulfillment could be validated as a unified psychological entity, aligning with one of the original objectives set by Loonstra et al. (2007).

M2 was a two-factor model in which self-acceptance forms one factor while self-actualization and self-transcendence combine into a second factor. This model addresses discrepancies in item allocation noted between the original EFS and its Chinese adaptation during EFA and is theoretically supported by Frankl’s (1962) assertion that self-actualization is often contingent upon self-transcendence, suggesting a potential linkage.

Model fit indices, summarized in Table 2, indicated that the three-factor model achieved the best fit, though the two-factor model was also acceptable, with both models meeting established criteria for goodness-of-fit.

**Table 2:** Confirmatory factor analysis fit index.

Model	*χ*/*df*	*CFI*	*TLI*	*RMSEA* (90 Percent C.I)	*SRMR*
1-factor (M1)	10.64	0.80	0.76	0.12 (0.11,0.12)	0.09
2-factor (M2)	4.82	0.92	0.90	0.07 (0.06,0.07)	0.06
3-factor (M3)	4.63	0.93	0.91	0.07 (0.06,0.08)	0.06

To further evaluate the structural validity of the three-factor model compared to the two-factor model, we analyzed the total scores, individual factor scores, and inter-factor correlations for each model. Findings are as follows:

In the three-factor model, the total scale score showed significant associations with self-acceptance (*r* = 0.62, *p* < 0.001), self-actualization (*r* = 0.89, *p* < 0.001), and self-transcendence (*r* = 0.85, *p* < 0.001). Self-acceptance correlated moderately with both self-actualization (*r* = 0.30, *p* < 0.001) and self-transcendence (*r* = 0.22, *p* < 0.001), while self-actualization demonstrated a strong correlation with self-transcendence (*r* = 0.76, *p* < 0.001).

As observed in the EFA, many items within the self-actualization and self-transcendence dimensions had high loadings on both factors, and the CFA fit indices further indicated that a two-factor model—where self-actualization and self-transcendence are combined into a single factor—was also viable. Additionally, correlation analyses among the three factors revealed a significant and high correlation between self-actualization and self-transcendence. Collectively, these findings suggest that, in the Chinese context, self-actualization and self-transcendence may function as a single factor. To further investigate, a bifactor model analysis was conducted for these two dimensions on Sample 4.

### 3.4 Evaluation of the bifactor model

To evaluate the bifactor model, we utilized two indices: explained common variance (ECV) and percent uncontaminated correlations (PUC) (Rodriguez et al., 2016a, 2016b). ECV measures the proportion of common variance attributed to both general and domain-specific factors. In this study, the general factor explained 85.2% of the common variance, with domain-specific factors accounting for the remaining 14.8%. This distribution suggests that the common variance is predominantly represented by the general factor. PUC, which measures the extent of EFS item correlations influenced by both general and domain-specific factors, yielded a value of 0.60. The high ECV combined with a moderate PUC indicates that many item correlations are primarily influenced by the general factor. This finding supports the notion that self-actualization and self-transcendence within the EFS may be treated as a unified dimension.

As previously noted, the constructs of self-actualization and self-transcendence reflect a fundamental human drive to explore and realize inherent potential while engaging meaningfully with the realities beyond oneself (Loonstra et al., 2007). This suggests that individuals achieving existential fulfillment continually strive to exceed their current capacities and self-concept. To encapsulate this ongoing developmental process within the Chinese context, we introduce the term “self-breakthrough” to represent this key factor in the Chinese EFS.

**Figure 1:**
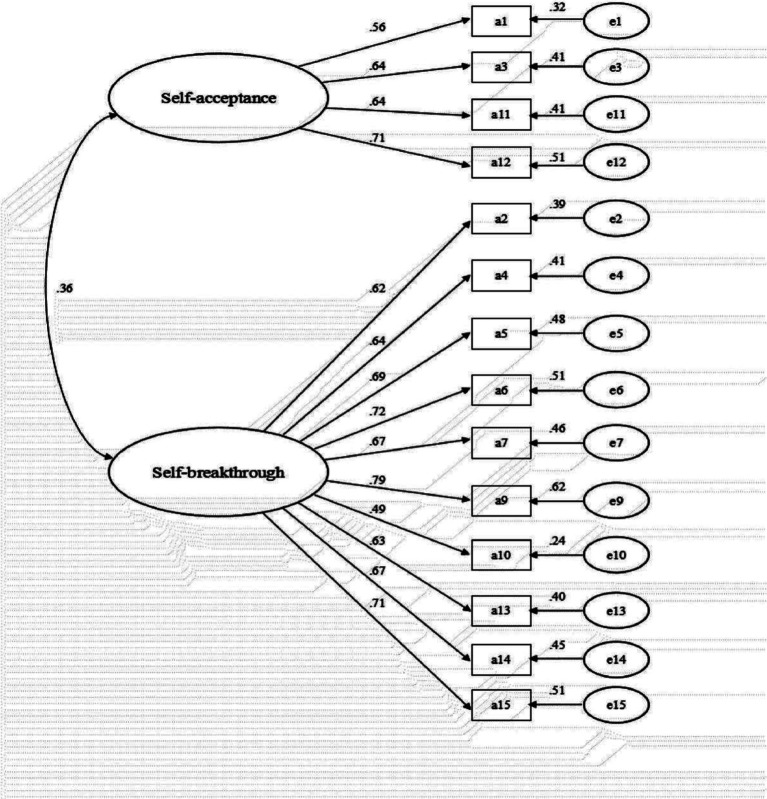
Confirmatory factor analysis for the Chinese EFS. Q1–Q15 represent items 1–15 in Table 1.

To provide more robust evidence for the construct validity of the scale, we conducted a CFA of the two-factor model, as depicted in Figure 1, using Sample 5. The results indicated that the CFI was 0.91, the TLI was 0.88, the RMSEA was 0.06 with a 90% confidence interval of 0.05–0.07, and the SRMR was 0.07. While the TLI fell slightly below the commonly accepted threshold of 0.90 for a good fit, all other fit indices were within ranges indicative of a well-fitting model. The results further reinforce the stability and structural validity of the two-factor Chinese version of the EFS.

In conclusion, the findings from these analyses indicate that the two-factor model of the Chinese EFS, comprising self-acceptance and Self-Breakthrough, provides the most robust structural validity.

### 3.5 Criterion-related validity test

To evaluate the criterion-related validity of the Chinese EFS, correlations were examined between the Chinese EFS and the MLQ, IWB, and SDS using data from Samples 5 and 6. The Chinese EFS showed a moderate positive correlation with the MLQ (*r* = 0.49, *p* < 0.001) and the IWB (*r* = 0.63, *p* < 0.001), and a moderate negative correlation with the SDS (*r* = −0.54, *p* < 0.001), confirming the scale’s criterion validity. See Table 3 for detailed correlations.

**Table 3:** Criterion-related validity of the Chinese version of EFS.

	Existential fulfillment	Self-acceptance	Self-breakthrough
**MLQ (*n* = 317)**	0.49**	0.15**	0.55**
Seeking of meaning	0.58**	0.24**	0.62**
Experiencing of meaning in life	0.28**	0.03**	0.34**
**IWB (*n* = 317)**	0.63**	0.54**	0.54**
General affective index	0.68**	0.55**	0.59**
Life satisfaction index	0.38**	0.16**	0.40**
**SDS (*n* = 33)**	−0.54**	−0.68**	**−0.34**
Psychotic-affective symptoms	−0.48**	−0.59**	−0.31
Somatic disorders	−0.34**	−0.60**	−0.13
Psychomotor disorders	−0.02	−0.07	−0.01
Psychological disorders of depression	−0.64**	−0.59**	−0.49**

The bolded numbers indicate the correlation coefficients between the total scores of the three criterion-related scales and the Chinese version of the EFS. **p < 0.01.

### 3.6 Reliability test

To assess the internal consistency of the scale, we first examined item-total correlations on Sample 4. Results showed that correlations between individual items and the total score ranged from 0.41 to 0.76, all of which exceeded the minimum criterion of 0.40 (Wu, 2010), confirming the homogeneity of the items across the scale.

Next, we employed CFA to assess the composite reliability (coefficient omega (*ω*)) of the total scale and subscales. Coefficient ω represents the proportion of variance in the total score attributable to all sources of common variance modeled within the scale. For the total scale, ω was 0.97. Regarding the two domain-specific factors, namely self-acceptance and self-breakthrough, *ω* was 0.89 and 0.96, respectively.

To further evaluate the reliability of the scale, we also assessed its internal consistency across Sample 5 and Sample 7. For Sample 5, the coefficient ω values for the total scale and the subscales of self-acceptance and self-breakthrough were 0.92, 0.87, and 0.91, respectively. For the initial test data of Sample 7, these coefficients were 0.97, 0.92, and 0.96, respectively. For the retest data of Sample 7, the coefficients were 0.96, 0.89, and 0.93, respectively. The cross-analysis of these samples suggests robust internal consistency reliability for both the overall scale and its subscales.

Additionally, test–retest reliability was assessed with a two-week interval on Sample 7, yielding values between 0.72 (*p* < 0.001) and 0.86 (*p* < 0.001) for both the total scale and its subscales, highlighting the scale’s strong temporal stability.

A comprehensive summary of these reliability results is presented in Table 4.

**Table 4:** Internal consistency reliability and retest reliability of the Chinese version of EFS.

	Existential fulfillment	Self-acceptance	Self-breakthrough
Internal consistency reliability (Sample 4)	0.97	0.89	0.96
Internal consistency reliability (Sample 5)	0.92	0.87	0.91
Internal consistency reliability (Sample 7, initial test)	0.97	0.92	0.96
Internal consistency reliability (Sample 7, retest)	0.96	0.89	0.93
Retest reliability (Sample 7, Two-week interval)	0.76	0.75	0.83

## 4 Discussion

In this research, the Chinese version of the EFS underwent its initial revision and cross-cultural validation, focusing specifically on Chinese college students. Through EFA, comparative analysis of multiple competing structural models using CFA, and examination of a two-factor model, this study found that, within the Chinese cultural context, the structure of the Chinese EFS diverges from the original three-dimensional model, reducing instead to two dimensions. Specifically, the dimensions of self-actualization and self-transcendence from the original scale have been combined into a single factor, which we have termed “self-breakthrough.” We propose that this cross-cultural difference in scale structure is related both to humanistic-existential psychology’s nuanced understanding of the relationship between self-actualization and self-transcendence and to the cultural perceptions of individual success and fulfillment in China.

One key rationale for this restructuring stems from divergent theoretical perspectives on self-transcendence. Humanistic-existential psychologists, such as Frankl (1962, 1963) and Maslow (1971), have noted that while self-actualization and self-transcendence can be conceptually distinct, they also overlap significantly. Frankl, for instance, suggested that the pursuit of meaning involves both reaching one’s potential (self-actualization) and transcending personal limitations (self-transcendence). Maslow (1971) similarly posited that self-actualization leads individuals to a deeper perception of reality and a capacity for profound interpersonal relationships, suggesting that self-actualized individuals often experience transcendence as they extend beyond themselves in meaningful relationships with others. Given this theoretical background, it is understandable that the distinction between self-actualization and self-transcendence is not always clear-cut, supporting their combination into a single factor within the Chinese context.

The second explanation for these structural adjustments is cultural context. In Eastern societies, particularly within Chinese culture, self-actualization is viewed as a socially oriented process, emphasizing the individual’s role responsibility and balance between personal and collective needs (Xu et al., 2017). This contrasts with the Western, more individualistic interpretation of self-actualization, which often emphasizes personal responsibility and the pursuit of individual goals and ideals (Maslow, 1971; Maehr, 1974). In Chinese culture, where collectivist ideologies are prevalent, self-actualization is considered incomplete without the support of the group, and self-transcendence is associated with individuals’ pursuit of future goals and ideals that often extend beyond their immediate realities (Yu and Yang, 1987; Yu, 2008). Thus, the structural differentiation in the Chinese EFS reflects both cultural specificity and cross-cultural theoretical perspectives. This study therefore indicates that a two-factor model is the optimal structure for measuring existential fulfillment in Chinese cultural settings.

Furthermore, this study found that the Chinese EFS outperforms the original scale regarding CFA fit indices. The original scale’s three-factor model yielded a CFI of 0.89 and an RMR of 0.08 (Loonstra et al., 2007), whereas our two-factor Chinese EFS achieved a CFI of 0.92 and an SRMR of 0.06. This improvement may be attributed to differences in the study populations. The original scale was tested on a more complex and heterogeneous sample of psychology graduates with an average age of 42.6, whereas our sample consists of Chinese undergraduate students, a relatively homogenous and less complex demographic. This difference in sample composition likely contributes to the observed variance in fit indices, supporting the suitability of the revised Chinese EFS for use in a younger, undergraduate context.

Consistent with our expectations, the Chinese EFS demonstrated moderate positive correlations with MLQ and IWB, supporting its criterion-related validity, along with a moderate negative correlation with SDS. These results suggest that higher EFS scores are associated with reduced depressive symptoms and increased levels of meaning and well-being, underscoring the scale’s robust criterion-related validity.

In terms of reliability, the Chinese EFS achieved an omega coefficient (*ω*) ranging from 0.87 to 0.97, indicating strong internal consistency. The test–retest reliability over a two-week interval was 0.76, with subscale values of 0.75 and 0.83. This temporal stability supports that the revised Chinese EFS meets high psychometric standards.

In summary, the Chinese EFS is a concise, effective tool for measuring existential fulfillment among Chinese college students, offering a reduced item set (14 items) that maintains clarity and minimizes respondent fatigue. Given its solid psychometric properties and cultural adaptability, the Chinese EFS stands to make a meaningful contribution to existential studies within Chinese populations.

### 4.1 Limitation and future directions

It is essential to acknowledge certain limitations in this research that require careful consideration. First, the study’s sample was drawn exclusively from universities in Fujian Province, potentially limiting the generalizability of the findings to the broader university-aged population in China. Fujian, located in southeastern China, has a moderately developed economy and a unique, relatively open yet conservative culture, which may affect the applicability of the study’s findings in other regions.

Secondly, despite efforts to recruit from various universities in Fujian, the limited number of top-tier institutions in the sample may not fully represent the diversity of the Chinese university landscape, potentially impacting the external validity of the results.

Thirdly, while the study provides initial evidence regarding the structure of existential fulfillment within a Chinese cultural context, it does not fully address the ongoing debate surrounding the specific connotations and structure of this construct. Although cultural influences were found to shape existential fulfillment, more in-depth exploration and validation of these cultural factors were beyond the scope of this study.

A significant limitation of the current study is the incomplete assessment of certain psychometric properties of the EFS. While this study offers preliminary evidence for the EFS’s use in a Chinese context, it did not rigorously evaluate key psychometric aspects such as discriminant validity and measurement invariance. Discriminant validity, which assesses the degree to which the EFS differs from related constructs, is crucial for establishing the unique role of existential fulfillment in psychological research. Similarly, measurement invariance, which tests whether the scale measures the same construct across different groups (e.g., gender or regional subgroups), is necessary to ensure the EFS’s applicability across diverse populations within China. Other aspects such as convergent validity, predictive validity, and known-group validity were also not thoroughly examined, which are important for establishing the EFS’s robustness.

Future research should aim to address these limitations by developing a locally adapted version of the EFS that reflects the unique connotations of existential fulfillment within Chinese culture. This process might involve conducting in-depth interviews to explore these cultural meanings, followed by coding techniques to inform the scale’s structure. A rigorous examination of measurement invariance and discriminant validity should be incorporated in future studies to confirm that the EFS provides consistent and distinct measures across various subgroups. Additionally, by employing a bottom-up approach in scale development, future research could yield a more nuanced and culturally sensitive tool, enhancing our understanding of existential fulfillment across China’s diverse populations. Such comprehensive psychometric evaluation would strengthen the EFS’s performance across varied contexts, thereby reinforcing its applicability in psychological research and assessment.

## Data Availability

The raw data supporting the conclusions of this article will be made available by the authors, without undue reservation.

## Footnotes

^1^ https://www.intestcom.org/files/guideline_test_adaptation_2ed.pdf
